# Successful Desensitization of a Patient with Rituximab Hypersensitivity

**DOI:** 10.1155/2015/524507

**Published:** 2015-01-20

**Authors:** Pinar Ataca, Erden Atilla, Resat Kendir, Sevim Bavbek, Muhit Ozcan

**Affiliations:** ^1^Department of Hematology, Ankara University, Cebeci, 06590 Ankara, Turkey; ^2^Department of Pulmonary Medicine, Immunology and Allergy Clinic, Ankara University, Cebeci, 06590 Ankara, Turkey

## Abstract

Rituximab is a monoclonal antibody which targets CD20 in B cells that is used for the treatment of CD20 positive oncologic and hematologic malignancies. Rituximab causes hypersensitivity reactions during infusions. The delay of treatment or loss of a highly efficient drug can be prevented by rapid drug desensitization method in patients who are allergic to rituximab. We report a low grade B cell non-Hodgkin lymphoma patient with rituximab hypersensitivity successfully treated with rapid drug desensitization. In experienced centers, drug desensitization is a novel modality to break through in case of hypersensitivity that should be considered.

## 1. Introduction

Monoclonal antibody research has entered a new era in targeting of specific proteins associated with disease pathogenesis [1]. However, hypersensitivity reactions to monoclonal antibodies limit their practicality [2]; these reactions have been reported following initial or repeated exposures [1]. Most of these reactions involve nonimmune cytokine releases that occur during the intravenous administration of the agent. IgE-related type I hypersensitivity reactions may also occur [3]. IgE-related mast cell activation promotes the release of histamine, leukotrienes, prostaglandins, proteases, and proteoglycans, which mediate early-type hypersensitivity reactions that can be accompanied by urticaria, shock, or even death [2].

If a patient develops hypersensitivity to a mandatory agent, drug desensitization should be applied. Desensitization is a treatment method that enables patients, who have previously experienced hypersensitivity reactions, to be treated with the culprit drug [1]. Desensitization is the rapid signal attenuation in response to stimulation on the other hand reduction in response to a drug after repeated administration defined as tolerance. Desensitization is effective for IgE-dependent or IgE-independent hypersensitivity reactions [4] but is contraindicated in patients with a history of Stevens-Johnson syndrome, toxic epidermal necrolysis, serum sickness, or hemolytic anemia [5].

Rituximab is a chimeric mouse-human anti-CD20 monoclonal antibody that is effective in non-Hodgkin's lymphoma (NHL), chronic lymphocytic lymphoma (CLL), rheumatoid arthritis, Wegener's granulomatosis, and microscopic polyangiitis [6]. In the rituximab prescription insert, the infusion-related reactions due to massive cytokine release are described as urticaria, hypotension, angioedema, hypoxia, bronchospasm, pulmonary infiltrates, acute respiratory distress syndrome, myocardial infarction, ventricular fibrillation, cardiogenic shock, anaphylactoid events, and death within 30–120 minutes of infusion [6]. The occurrence of an infusion reaction in NHL is reported to be 77% [6]. However, 5% to 10% of the reactions to rituximab are considered as immediate type I hypersensitivity [3]. Studies demonstrated that the prolongation of treatment should be managed by rapid drug desensitization in patients who are allergic to rituximab [7, 8]. Rapid desensitization allows safe readministration of a medication after certain types of immediate hypersensitivity. However, desensitization protocols for monoclonal agents are followed in few centers, and most researchers are unaware of the involved methods. Therefore, we present a patient with NHL who was treated successfully with rituximab in our center despite having a history of severe rituximab related adverse reaction.

## 2. Case Presentation

A 54-year-old male was admitted to the gastroenterology clinic with epigastric pain, weight loss of 6 kg, night sweats, and high fever that started a month prior to admission. He reported no severe allergic reactions in medical history; however, he described flushing and flu-like symptoms during gardening. In his physical examination, we detected nonmobile pathologically lymphadenopathies in bilateral cervical, axillary and inguinal regions, and splenomegaly. His routine laboratory results were as follows: lactate dehydrogenase: 133 U/L, Beta2 microglobulin: 4.246 mg/dL, leucocytes: 5.6 × 10^9^/L, erythrocytes: 3.92 × 10^12^/L, platelets: 137 × 10^9^/L, and hemoglobin: 12.6 g/dL. In his cervical and inguinal ultrasonography and thoracoabdominal computed tomography (CT) scan, bilateral axillary, mediastinal, hilar, paraceliac, peripancreatic, portal hepatogastric, and inguinal pathological lymphadenopathies were detected. His right axillary region lymph node biopsy and bone marrow biopsy results indicated low-grade B cell (follicular) NHL. We diagnosed him with Stage 4 disease and prescribed 6 cycles of an R-CHOP (rituximab 375 mg/m^2^, cyclophosphamide 750 mg/m^2^, adriamycin 50 mg/m^2^, vincristine 1.4 mg/m^2^ (max: 2 mg), and prednisone 100 mg/day) protocol. Prior to the first dose of rituximab, 45.5 mg of pheniramine (intravenous) and 500 mg of acetaminophen (peroral) were administered. The patient developed flushing within 5 minutes following administration. The infusion was then interrupted, and 45.5 mg of pheniramine was administered. The infusion was slowly restarted. Ten minutes following readministration, generalized urticaria, dyspnea, and nausea developed. His physical examination revealed the following: 38.2°C body temperature, arterial blood pressure of 100/60 mmHg (initial arterial blood pressure of 120/80 mmHg), arterial O_2_ saturation: 92%, 120 beats/min tachycardia, and bilateral rhonchi with no stridor or pharynx edema. The infusion was stopped and the reaction was treated with the administration of 100 mg methylprednisolone and pheniramine. The tryptase level was 24.60 *μ*g/L (*n*: <11 *μ*g/L) at four hours after the hypersensitivity reaction. Next day, the patient received CHOP only protocol (no rituximab) uneventfully. We referred the patient to the Immunology and Allergy Clinic to continue rituximab treatment.

In the Immunology and Allergy Clinic, his reaction was defined as Grade 3 according to the Brown Classification, which indicates a severe systemic hypersensitivity reaction [9]. A 12-step rapid drug desensitization protocol was planned for the patient. He was premedicated with H1 and H2 blockers with systemic steroids and was desensitized by an experienced allergist and nurses according to established protocols (Table 1). The rituximab dosage was not decreased throughout the following cycles. The basal tryptase level was 8.89 *μ*g/L. At the twelfth step of the protocol, he developed urticaria in his face and body; thus, desensitization was interrupted and treatment was administered. The infusion was restarted without adverse effects. He had a positive skin prick test result for rituximab before second course. During further cycles, the same dose rituximab was administered with minimal urticaria in his face and body so desensitization protocols were completed with increased premedications. The patient is followed by complete response after 6 cycles of R-CHOP; the bone marrow involvement was disappeared. The patient's desensitization protocol is currently ongoing during maintenance therapy.

## 3. Discussion

Rituximab is a chimeric monoclonal antibody, changed the natural history of some catastrophic disorders, directed to the CD20 molecule and currently used in treatment of lymphoproliferative diseases and several rheumatologic disorders. Intentionally, rituximab is one of the most frequent causes of acute infusion reactions due to massive cytokine release [10]. Clinical manifestations of IgE-related and non-IgE related infusion reactions overlap; rash, hypotension, nausea, tachycardia, and shortness of breath have been described in both reactions [3]. The severity of infusion reactions is associated with high tumor burden or advanced disease which usually occurs after the first administration of rituximab [11]. Although our patient had high tumor burden, elevated tryptase levels, positive skin test supported the hypersensitivity reaction rather than infusion reaction.

Monoclonal antibodies have increasingly been used in routine practice; thus, hypersensitivity reactions are becoming increasingly more common. Recent studies reported the presence of serum anti-drug antibodies in pretreated patients [10]. Anti-drug antibodies are mostly IgG and IgE which shows the adaptive immune response to the drug [12]. Castells et al. reported 413 cases of desensitization in 98 patients for reactions to carboplatin, cisplatin, oxaliplatin, paclitaxel, liposomal doxorubicin, doxorubicin, and rituximab. A twelve-step rituximab desensitization protocol was performed seven times in three of the 98 patients. Two of these patients were diagnosed with lymphoma and one was diagnosed with polymyositis. Two patients with lymphoma had rashes and pruritus, while the third patient experienced syncope. Similar to our case, the infusion rate was decreased; however, there were no changes in the hypersensitivity reactions. Regression due to the reduction of the infusion rate could indicate immune-related or non-IgE-related hypersensitivity reactions [4].

The same researchers performed another study of 23 patients with 105 cases of desensitization. A total of 14 patients with no prior monoclonal antibody exposure developed (primarily) intermediate-grade hypersensitivity to rituximab. Eleven of the 14 patients had a reaction during the first administration that was similar to our case, while 1 patient experienced a reaction during his third administration, 1 patient during her fourth administration, and another patient during repeated administrations. None of the patients in the study exhibited skin prick test positivity with rituximab; however, 6 of the 9 tested patients had intradermal skin test positivity [3]. To prevent false-negative results, skin tests should be administered two weeks after a reaction; however, if the waiting period would interrupt the patient's treatment, the desensitization protocol should be performed first [13]. We desensitized the patient prior to the skin test; however, before second course the skin test was positive. The increase in the tryptase level in our patient indicates a hypersensitivity reaction due to IgE. In rapid drug desensitization protocols, the drug is administered in small increments [1]. The goal of this method is to decrease the mast cell and basophil response to the drug [14]. Rapid drug desensitization precipitates transient unresponsiveness; thus, the patient should be desensitized again during each exposure [2].

Using rapid drug desensitization protocols, it is possible to continue monoclonal antibody administration after hypersensitivity reactions. As a result, early hypersensitivity reactions to rituximab can be managed in appropriately trained centers via rapid drug desensitization to enable rituximab continuation with transient tolerance. By this, an important drug, rituximab, which is opening a new era in management of hematological malignancies, can be prevented from early cessation.

## Figures and Tables

**Table 1:** Rituximab desensitization protocol.

Step	Solution	Rate (mL/h)	Time (min)	Dose	Total dose
1	1	2.0	15	0.015	
2	1	5.0	15	0.037	
3	1	10.0	15	0.075	
4	1	20.0	15	0.150	0.277
5	2	5.0	15	0.375	
6	2	10.0	15	0.750	
7	2	20.0	15	1.500	
8	2	40.0	15	3.000	5.625 (1–8: 5.902) 750–5.902: 744 mg will be given in next steps
9	3	10.0	15		
10	3	20.0	15		
11	3	40.0	15		
12	3	75.0	240		

Solution 1: 250 mL, 5% dextrose, /0.8 mL  rituximab (0.03 mg/mL: 1 : 100 of total dose, 250 mL/7.5 mg).

Solution 2: 250 mL, 5% dextrose, /7.5 mL rituximab (0.30 mg/mL: 1 : 10 of total dose, 250 mL/75 mg).

Solution 3: 250 mL, 5% dextrose, /74.4 mL rituximab.

Premedication: 20 minutes before pheniramine 45.5 mg IV, prednisolone 100 mg IV, and famotidine 20 mg IV.
